# Three-Dimensional Image of Cleavage Bodies in Nuclei Is Configured Using Gas Cluster Ion Beam with Time-of-Flight Secondary Ion Mass Spectrometry

**DOI:** 10.1038/srep10000

**Published:** 2015-05-11

**Authors:** Noritaka Masaki, Itsuko Ishizaki, Takahiro Hayasaka, Gregory L. Fisher, Noriaki Sanada, Hideo Yokota, Mitsutoshi Setou

**Affiliations:** 1 Dept of Cell Biology and Anatomy, Hamamatsu University School of Medicine, 1-20-1 Handayama, Higashi-ku, Hamamatsu, Shizuoka 431-3192, Japan; 2 ULVAC-PHI, 370 Enzo, Chigasaki, Kanagawa 253-8522, Japan; 3 Physical Electronics, 18725 Lake Drive East, Chanhassen, MN 55317, USA; 4 Image Processing Research Team, Center for Advanced Photonics, RIKEN, 2-1 Hirosawa, Wako, Saitama 351-0198, Japan

## Abstract

Structural variations of DNA in nuclei are deeply related with development, aging, and diseases through transcriptional regulation. In order to bare cross sections of samples maintaining sub-micron structures, an Ar_2500_^+^-gas cluster ion beam (GCIB) sputter was recently engineered. By introducing GCIB sputter to time-of-flight secondary ion mass spectrometry (TOF-SIMS), we analyzed the 3D configuration and chemical composition of subnuclear structures of pyramidal cells in the CA2 region in mouse brain hippocampus. Depth profiles of chemicals were analyzed as 3D distributions by combining topographic analyses. Signals corresponding to anions such as CN^−^ and PO_3_^−^ were distributed characteristically in the shape of cell organelles. CN^−^ signals overlapped DAPI fluorescence signals corresponding to nuclei. The clusters shown by PO_3_^−^ and those of adenine ions were colocalized inside nuclei revealed by the 3D reconstruction. Taking into account their size and their number in each nucleus, those clusters could be in the cleavage bodies, which are a kind of intranuclear structure.

The three-dimensional configuration of a nucleus, which is characterized as containing one-dimensional information on a DNA sequence^1^,^2^, is thought to be a key to epigenetic alterations, as variations in configuration cause different phenotypes in monoclonal populations through transcriptional diversity^3^,^4^,^5^. Structural variations of a genome are dependent on DNA methylation and histone modifications^6^, as well as on enhancer-promoter interaction^7^; thus, such variations relate to development, aging, and diseases^8^. Structural variations in cells and their roles in transcription modifications have been analyzed using 4C assays^9^ and Chip-Seq^10^. The processing of transcripts by subnuclear structures, such as nuclear speckles and cleavage bodies, also plays important roles in epigenetic alterations^3^,^11^. Various imaging methods, such as sheet illumination microscopy using a Bessel beam^12^, dual-view plane illumination^13^, and the use of reflect light sheets^14^, have been developed for the direct observation of 3D structures of nuclei as common interests in biology^15^. Direct measurement of chemicals at sub-micrometer resolution is one of the next big requirements in biological study. Compared with recent improvements in lateral resolution, resolution for depth direction remains hindered by diffraction and the extra-focal illumination of the laser^16^. Observation of a 3D structure using electron microscopy, which has the highest lateral resolution, requires improvement for sectioning without disrupting the microscopic structure^17^. The precise loci of targets are also obscured by labeling using antibodies^18^.

Secondary ion mass spectrometry (SIMS) is one of the most popular imaging mass spectrometry (IMS) techniques. With the use of a tightly focused ion beam, it has the highest spatial resolution, beyond even the optical limitation of a laser focus^19^. SIMS has been used for element and isotope analyses^20^,^21^ as a powerful tool to quantify and visualize known molecules on the scale of tens of nanometers^22^, while the identification of newly found molecules by MS/MS analysis is still a challenge for SIMS^23^. Precise isotope ratio measurements can reveal even chemical processes underlying mechanisms^24^. Among the various types of SIMS analyses, time-of-flight (TOF) SIMS can detect numerous signals simultaneously from a wide range of mass-to-charge ratios, described as *m/z*. Recently, single-cell-level IMS measurements are required to characterize cell types and to provide explicit evidence to distinguish them^23^. To achieve this, the primary ion beam needs to be improved to ionize larger molecules, and thus careful examination from the aspect of analytical chemistry is also required.

Depth profiles and 3D reconstructions are the most suitable applications for TOF-SIMS analyses^25^, as they can perform alternating or sequential measurements of the same region. In a SIMS analysis, only an area with a thickness of less than 10 nanometers from the surface of the sample is analyzed^26^, and deeper parts can be maintained undisturbed by using appropriate acceleration energy depending on the type of ion beam^27^. Even compared with fluorescence microscopic observations^16^, TOF-SIMS provides a more precise resolution in the *z*-axis^25^. Recently, the 3D subcellular structure of HeLa cells was revealed^28^,^29^. By combining a second ion beam, 3D TOF-SIMS can be performed rapidly and obtain signals effectively^30^. For this purpose, a gas cluster ion beam (GCIB)^31^ was recently engineered to prevent surface molecular fragmentation of biological samples and to remove the damaged layer caused by the analysis beam^32^,^33^. GCIB can now be combined with TOF-SIMS to visualize the 3D ion distribution of a single cell.

In the present study, we propose an analytical procedure for 3D configurations in a single cell nucleus using GCIB-TOF-SIMS. To elucidate the 3D distributions of biomolecules reflecting nuclear bodies, we used GCIB for sputtering to expose a flattened region during depth analysis. Our method demonstrated a better spatial resolution of less than 200 nm and successfully showed 3D configurations of nuclei and distinguishable subnuclear structures that can tentatively be identified as cleavage bodies.

## Results

### Estimation of depth with the sputter rate

**Figure 1:**
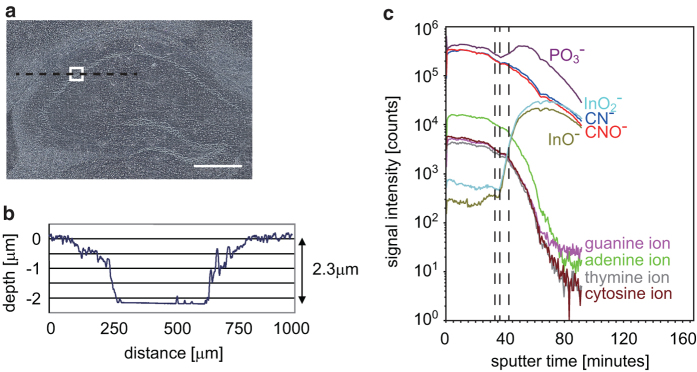
Topographic profile and depth analysis. A bright-field image of a sagittal section of mouse brain (**a**). The region delimited by the white line was analyzed. Scale bar: 400 μm. A topographic profile after the TOF-SIMS analysis (**b**). The scan was performed on the black dashed line shown in (**a**), and the precise depth was measured as 2.3 μm ± 0.11 (mean ± S.E.M.) from 3 scans. The total counts of various ions obtained at each sputter time are plotted (**c**).

First, we optimized the acceleration energy of the GCIB sputter to 10 kV 4 nA, as this condition was suitable to bare flattened cross sections with relatively fast sputter rates. The thickness of a sample section was determined as 10 μm, taking cell size and measurement time into account. To analyze similar cells effectively, we selected the CA2 region, a small region between CA1 and CA3 along the S-curve of the mouse brain hippocampus, because pyramidal cells of suitable size for our experiment are closely packed in this region. The region we analyzed is delimited by a white line in a bright-field image of the mouse brain section in Fig. 1a.

We performed TOF-SIMS measurement after GCIB sputtering for this region and obtained 3D high-resolution chemical maps. After TOF-SIMS analysis, we measured the topographic profile by Stylus Profiler. The profile shown in Fig. 1b exhibits a straight bottom line at a distance between 250 μm and 650 μm, reflecting the glass slide surface. From tree profiles, the sample thickness was estimated as approximately 2.3 μm ± 0.11 (mean ± S.E.M.).

Next, to determine when the sputtering reached the glass slide, we analyzed the depth profile of ion intensity for various ions^25^,^34^,^35^. The ion intensities were integrated over the measured region at each sputter time. The results are shown in Fig. 1c. We plotted the integrated intensities for several typical ions against the sputter time. The signals at *m/z* 26.0, *m/z* 42.0, and *m/z* 79.0 represent CN^−^, CNO^−^, and PO_3_^−^, respectively^29^,^36^. Signals at *m/z* 110.0, 125.0, 134.1, and 150.1 were considered cytosine, thymine, adenine, and guanine by comparison with mass spectra obtained from standard samples (data not shown) and reported values^37^. The intensities of these signals showed a plateau by 33 min and then started to decrease. Conversely, signals of InO^−^ (*m/z* 131.0) and InO_2_^−^ (*m/z* 147.0), used to coat the glass slides, showed remarkable increases after about 36 min. As the intensities reached the half-maximal point at 42 min, we decided that the sputter reached the glass surface at that time point. Using these quantities, we estimated the sputter rate as 55 nm/min in this experiment.

### Mass spectrum obtained from entire sample

**Figure 2:**
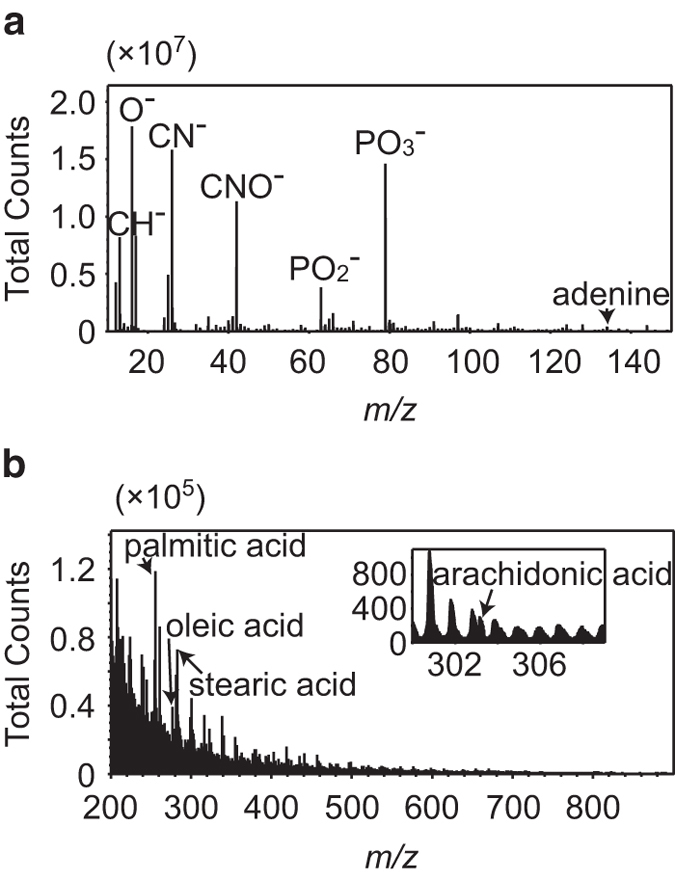
Mass spectrum of GCIB-TOF-SIMS. An enlargement of mass spectrum *m/z* from 10 to 150, where the most abundant signals were concentrated, is shown in (**a**). An enlargement of *m/z* between 200 and 900 is shown in (**b**). Signals of fatty acids are indicated using arrows; palmitic acid (*m/z* 255.2), oleic acid (*m/z* 281.2), stearic acid (*m/z* 283.2), and arachidonic acid (*m/z* 303.3). Inset: the enlargement of the mass spectrum around the arachidonic acid signal.

We obtained the integrated mass spectrum in the negative ion mode from *m/z* 0 to 1850 after 42 min. As most of the signals are assembled below *m/z* 150, a resizing of *m/z* from 10 to 150 is shown in Fig. 2a. Various peaks were found and identified according to the previous reports^29^,^36^, as follows: *m/z* 13.0 as CH^−^, *m/z* 16.0 as O^−^, *m/z* 26.0 as CN^−^, *m/z* 42.0 as CNO^−^, *m/z* 63.0 as PO_2_^−^, and *m/z* 79.0 as PO_3_^−^. Looking at the *m/z* region from 100 to 150, we also found peaks we thought reflected nucleic acids, e.g., *m/z* 134.1 as adenine. Other nucleobases—thymine, guanine, cytosine, and uracil—were also detected, but their intensities were not strong enough to discuss as distinct distributions (Fig. S1). As shown in Fig. 2b, we also focused on a larger *m/z* region from 200 to 900, where various metabolites and phospholipids were detected such as by matrix-assisted laser desorption/ionization IMS. The peaks corresponded to fatty acids (FA) palmitic acid (*m/z* 255.2; PA), oleic acid (*m/z* 281.2; OA), stearic acid (*m/z* 283.2; SA), and arachidonic acid (*m/z* 303.3; AA), according to previous reports^37^,^38^,^39^. Enlargement of the spectrum around the AA signal is shown as an inset in Fig. 2b.

### Ion image for each depth plane

**Figure 3:**
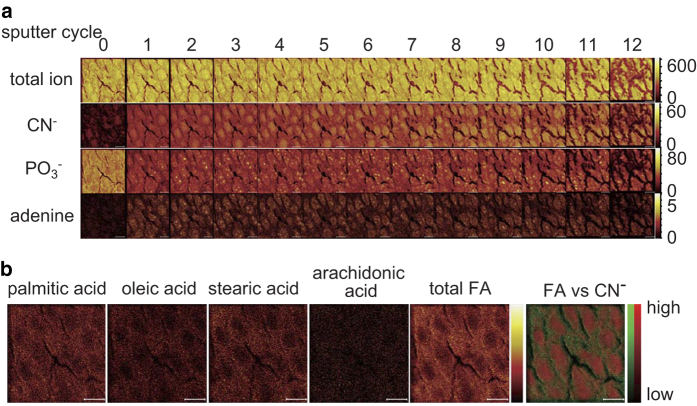
Ion images of GCIB-TOF-SIMS. Ion images at all depth planes are shown in (**a**). Scale bar: 10 μm. The ion images at sputter cycle 0 show an ion distribution on the surface of the section. All of these ions show spatial inhomogeneity indicating subcellular structures. Ion images corresponding to fatty acids (FAs) were reconstructed by gathering signals from all depth planes, and the results are shown in (**b**). Ion images of palmitic acid, oleic acid, stearic acid, arachidonic acid, and the total of these signals are illustrated in relative intensity scale. Maximum ion counts in the scale were 12 (palmitic acid), 9 (stearic acid), 7 (oleic acid), 4 (arachidonic acid), and 17 (total FAs). The distribution of the signal of total FAs, shown in green, is compared with the z-projection signals from CN^−^, shown in red.

To analyze the 3D distribution of molecules, we reconstructed an ion image for each sputter cycle, i.e., at every 192.5 nm depth plane. Typical images using a pseudocolor scale are shown in Fig. 3a. The ion images reconstructed using total ions illustrated densely packed cell bodies. The ion images reconstructed from CN^−^, which showed high intensity in the mass spectrum, showed the largest single cluster inside the cells. We also found small clusters shown by PO_3_^−^ signals and adenine ion signals. PO_3_^−^ signals at sputter cycle 0 appeared uniform, similar to the total ion image. After the sputtering, small clustered signals of PO_3_^−^ were unveiled inside the cells. Ion images reconstructed from adenine signals also showed small clusters, and they colocalized with PO_3_^−^ signals, while 38% of PO_3_− clusters did not contain adenine ion.

Ion images corresponding to FAs were reconstructed by gathering signals from the entire depth plane, and the results are shown in Fig. 3b. Ion images of PA, OA, SA, AA, and the total of these FAs were reconstructed and shown in relative scales. The maximum ion counts for the respective scales were 12 (PA), 9 (SA), 7 (OA), 4 (AA), and 17 (total FAs). The distribution of the total FAs signal (green) are compared with z-projection signals from CN^−^ (red). These signals appeared to be complementary.

### Analysis of 3D ion distributions by reconstructing 3D objects

**Figure 4:**
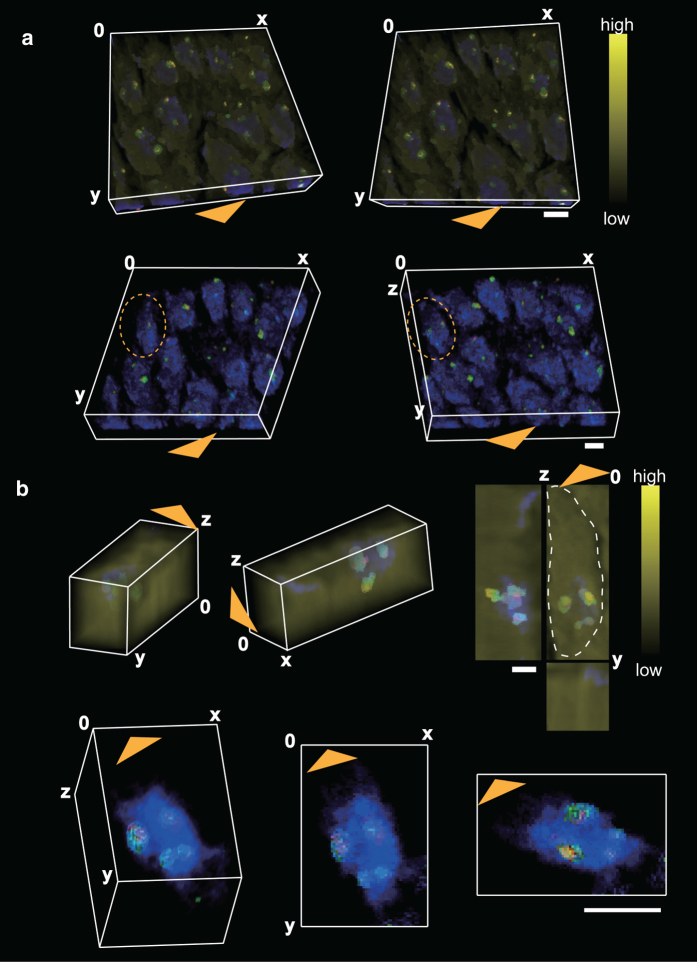
3D objects reconstructed from ion images. Snapshots of entire binarized images of CN^−^ (blue), PO_3_^−^ (green), and adenine ion (red) are shown with the total ion signal (yellow) in the upper panels of (**a**). Snapshots without the total ion signal are also shown in the lower panels of (**a**). To elucidate the loci of these clusters inside a cell, a single cell was cropped (**b**). An expanded view of this cube is also shown, with the cell shape delimited by a white dash line. Enlarged snapshots without the cell body are shown in the lower panels of (**b**). In every figure, the white lines schematically represent the orthogonal coordinate system, the orange arrowheads indicate the direction of apical dendrites, and all the scale bars represent 5 μm.

To compare the loci of these clusters in detail, we reconstructed 3D objects from the binarized ion images. Snapshots of the results are shown in the upper panels of Fig. 4a. Cell bodies are also shown using relative total ion intensities in a yellow pseudocolor scale. Clusters from CN^−^ signals are shown in blue, clusters from PO_3_− in green, and clusters from adenine ions in red. To improve visualization, we expanded the sample thickness to the originally sectioned thickness by multiplying by 4.35, and we ignored PO_3_− accumulation at the sample surface, sputter cycle 0, to avoid the possibility of an artifact such as cholesterol migration^36^. The orthogonal coordinate is shown using white lines, and the scale bar represents 5 μm. Snapshots without cell bodies are also shown in the lower panels of Fig. 4a.

To observe the locus of these clusters inside a single cell, we cropped a cell and indicated its location within an orange dashed line. Merged snapshots including the cell body are shown in upper panels of Fig. 4b. An expanded view of this cube is shown with a white dashed line drawn around the cell. The cell body is also visualized using the total ion signal with 8-bit yellow scale. It is clear that localization of all ion clusters is concentrated at the main part of the cell body. Furthermore, only one CN^−^-rich region is included in the cell body, while multiple clusters of the PO_3_− and adenine ions are included. To investigate the positional relationships of these clusters, snapshots of the single CN^−^-rich region are shown together with PO_3_− and adenine ion clusters in the lower panels of Fig. 4b. We found that most of the PO_3_− and adenine clusters were in CN^−^-rich regions. Moreover, most of the adenine clusters were colocalized with PO_3_− clusters, which are shown in light blue. Five totally colocalized clusters can be seen inside the CN^−^-rich region in the lower panels of Fig. 4b. In every snapshot, orange arrowheads indicate the direction of the apical dendrites. Rotating views of these objects shown in Fig. 4 are presented as supporting movies S1, 2, 3, 4.

### Comparison of ion imaging with fluorescence imaging

**Figure 5:**
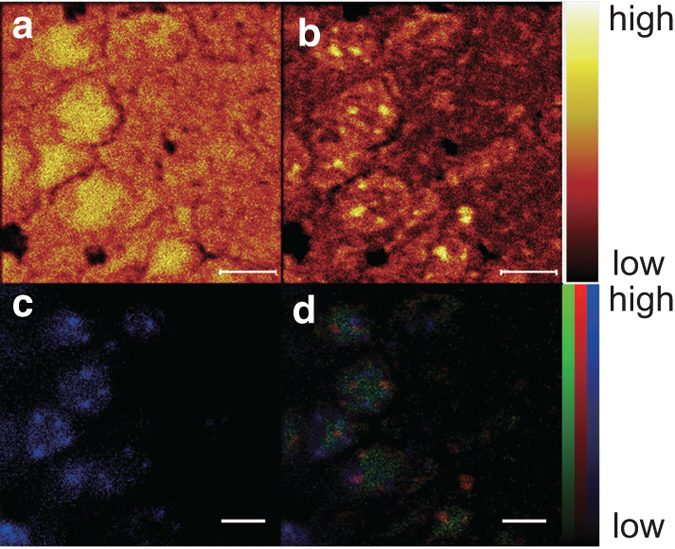
Comparison of CN^−^ and PO_3_^−^ signals with DAPI fluorescence. After GCIB sputtering, a CN^−^ image (**a**) and a PO_3_− image (**b**) were obtained. DAPI fluorescence was observed at the same position as shown in (**c**). Overlay of DAPI fluorescence (blue) onto ion images (CN^−^: green, PO_3_−: red) is shown in (**d**). Scale bar: 5 μm.

To determine whether the CN^−^-rich regions represented nuclei, we performed DAPI staining of the sections analyzed using TOF-SIMS. We sputtered samples and then obtained ion images of CN^−^ and PO_3_− (Fig. 5a,b). After the TOF-SIMS measurement, we stained with DAPI and observed the sample using an epifluorescence microscope at exactly the same position. The result is shown in Fig. 5c. To compare the distribution of DAPI fluorescence with CN^−^ and PO_3_− signals, we merged these images (Fig. 5d). The relative intensity of DAPI fluorescence is shown in blue, and the relative ion intensities of CN^−^ and PO_3_^−^ are shown in green and red, respectively. The distributions of DAPI fluorescence and CN^−^ signal matched well, and thus we believe the CN^−^-rich regions reflected the nuclei. It must be noted that DAPI fluorescence covered larger areas than the CN^−^-rich regions observed by TOF-SIMS. That is why DAPI fluorescence showed a z-projection of all depth planes while CN^−^ originated from a single plane.

### Cluster size distribution

**Figure 6:**
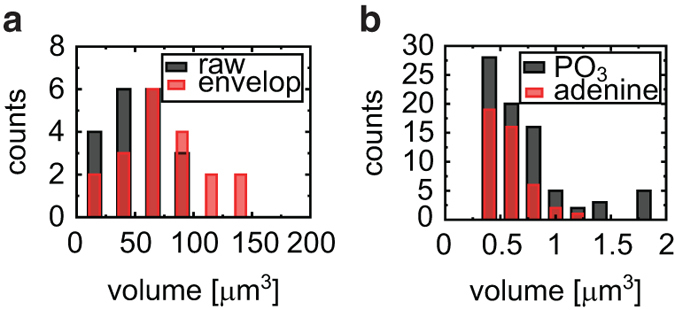
Volume distribution of 3D objects. The volume distribution of CN^−^-rich regions (black) and that of their envelopes (red) are shown in (**a**). The averages were 50.4 ± 25.0 μm^3^ for raw volume and 72.1 ± 34.4 μm^3^ for envelope volume. The volume distributions for PO_3_− (black) and adenine clusters (red) are also shown as histograms in (**b**). The average volumes are 0.70 ± 0.53 μm^3^ for PO_3_− clusters and 0.43 ± 0.28 μm^3^ for adenine clusters, respectively.

To characterize the clusters quantitatively, we analyzed the number and size of these clusters. We estimated the volume of a single voxel as follows: the area of the pixel in each ion image is (50 μm/256 pixels)^2^, and the distance of each depth plane is approximately 2.3 μm/13 cycles; therefore, the volume of a single voxel is calculated as 0.0073 μm^3^. The result for CN^−^ is shown in Fig. 6a. To compare the result with those of optical microscopic studies, we also calculated the volumes of envelopes, which include cavities contained within CN^−^-rich regions. We analyzed a total of 21 CN^−^-rich regions and found that a monomodal size distribution. The average and standard deviations for raw volume and envelope volume in our results were calculated as 50.4 ± 25.0 μm^3^ and 72.1 ± 34.4 μm^3^, respectively. Assuming that these volumes were spherical, the average diameters were calculated as 4.6 μm for raw volume and 5.2 μm for envelope volume.

Similarly, we also analyzed a total of 73 PO_3_− clusters and 45 adenine clusters, and the results are shown together in Fig. 6b. Both distributions appeared similarly below 1.25 μm^3^. Upon visual inspection, the size distribution of the PO_3_− clusters seemed to be bimodal, whereas the distribution of the adenine clusters appeared monomodal. The volume of these clusters also varied widely, and the averages of these clusters were obtained as 0.70 ± 0.53 μm^3^ for PO_3_− and 0.43 ± 0.28 μm^3^ for adenine ions. All of the PO_3_− clusters and adenine clusters are located inside the nuclei, and about three PO_3_^−^ clusters and two adenine clusters are present in a single nucleus on average. Assuming a spherical shape, their diameters were calculated as 1.1 μm for PO_3_− and 1.0 μm for adenine ion.

## Discussion

To convert voxels to physical volumes, we measured precisely the sample thickness and sputter cycles to reach the glass slide (Fig. 1). In our experiment, sample thickness after dehydration was 2.3 μm. This is about twice the thickness of the rat brain section used by Bich *et al*.^30^. Their section was compressed to 1/12 of the 25 μm originally sectioned thickness, while ours was compressed nearly to 1/4, from 10 μm to 2.3 μm. Therefore, the section used in our study was more suitable for 3D analyses about the z direction.

The integrated secondary ion mass spectrum obtained from sample tissue showed various peaks reflecting biomolecules, including FAs (Fig. 2). On the other hand, few signals corresponding to raw phospholipids were found. Taking into account that free FAs are known to be few and phospholipids are known to be abundant^40^, we considered that in-source decay changed the ratio. This result is consistent with those of the earlier SIMS studies^41^,^42^,^43^. Palmitic acid (PA), oleic acid (OA), stearic acid (SA), and arachidonic acid (AA) are commonly detected FAs in mouse brain by methods such as gas chromatography (GC)^44^,^45^. The abundance ratios of these FAs in our present study are almost identical to those in the earlier result^45^.

We found CN^−^-rich regions and clusters formed by PO_3_− and adenine ions, respectively, from the ion images (Fig. 3). Various biomolecules are considered sources of PO_3_−, such as phospholipids, nucleic acids, and ribonucleotides. Because PO_3_− is also transferred, bonded, and dissociated in various biological processes, e.g., phosphorylation, PO_3_− is an important molecule for living organisms. Analysis of its distribution is also important. The presence of PO_3_− in nuclei is consistent with the results of previous reports using C_60_ sputter^28^,^29^. Compared to the C_60_ result, GCIB sputtering could provide higher spatial resolution and thus show the cluster-type structure of PO_3_−. The presence of adenine in nuclei is also consistent with an earlier report in which C_60_ was used as the primary ion. Moreover, by decreasing the dynamic ranges of ion images, we found that the distribution of PO_3_− was similar to that of the CN^−^-rich region, consistent with earlier reports (Fig. S2). As the CN^−^-rich region appeared complementary with FAs, PO_3_− are considered to come from nucleobases rather than phospholipids. On the other hand, the distribution of each FA shown in Fig. 3b was almost uniform in cytosol. Recent lipidomic studies figured out the importance of FA distribution with different carbon chain lengths and double bonds, depending on their biological roles^40^. Thus, it was expected that FAs also exhibited some kind of tiny structures such as endoplasmic reticulum. Our study reveals similarities in the distribution of PA, OA, SA, and AA. These FAs are complementary with the z-projected CN^−^-rich region, as seen in the image of all FAs. FAs are therefore considered to come mainly from cytosol.

As pyramidal cells are directionally arranged along CA regions associated with the anatomical structure of the hippocampus, their polarity is determined by the direction toward apical dendrites and basal dendrites^46^,^47^. The spatial distribution of PO_3_− clusters and adenine clusters in the CN^−^-rich region seems to be irrespective of the direction of apical dendrites, as shown in Fig. 4. Thus, the polarity of the cell body is considered not to affect the distribution of these clusters in the nuclei. Adenine ions may come from ribosome or nucleic acids in the nucleus. On the other hand, since the polyadenylation of RNA is known as a main role of cleavage bodies^48^, the clustering of adenine in cleavage bodies is reasonable. Accumulations of polyadenylated mRNA inside nuclei were also observed at the CA1 region in adult rat hippocampus by in situ hybridization^49^; our results are consistent with those of that report. The colocalization of adenine with PO_3_− also evokes a ribonucleotide such as adenosine monophosphate (AMP), adenosine diphosphate (ADP), or adenosine triphosphate (ATP). As cleavage bodies are known to colocalize with ATP-dependent RNA helicase DDX1^50^, the clustering of PO_3_− also suggested a cleavage body.

We confirmed the CN^−^-rich region overlapping the nuclei by comparing the loci with DAPI fluorescence, as shown in Fig. 5. As it is known that the nitrogen-to-carbon ratio of nucleic acids is nearly 1.5 times higher than the ratio of proteins^51^, CN^−^ signals were considered to be enriched in nuclei. Subcellular analysis of human osteogenic sarcoma cells and human astrocytoma cells using a Raman spectroscope has revealed that Raman shift by the CN double bond was not remarkable in both cytosol and nuclei^52^. This is because the signal from the carbon-nitrogen bond in the Raman spectrum contained both CN^−^ and CNO^−^. In the present study, CNO^−^ was found mainly in cytosol complementary with CN^−^ (data not shown). Consistently, the signal of CN^−^ + CNO^−^ was found all over the cell body in the earlier report^29^. Our modality has an advantage in that the distribution of CN^−^ and the distribution of CNO^−^ can be clearly distinguished.

Many aspects of the substance of intranuclear structures, such as subnuclear bodies and nuclear speckles, remain unclear, as it is difficult to purify and separate such tiny structures. Taking the results of previous reports^53^,^54^ into account, we think that the intranuclear structures we visualized are nucleoli or cleavage bodies, as their size and number in each nucleus shown in Fig. 6 are consistent with those previously reported^53^,^55^. The 3D shapes of cleavage bodies were not only spherical but also long thin, which we consider to reflect the cleavage bodies processing RNA^56^. GCIB-TOF-SIMS can figure out both shapes, while fluorescence observation requires different labeling. The difference between the number of PO_3_− clusters and that of adenine ion clusters is also consistent with the previous result that RNA is absent in 20% of cleavage bodies^55^.

Aiming at submicron 3D analysis, we combined GCIB sputtering and TOF-SIMS measurement. GCIB bares cross-sections of samples while maintaining microscopic structures without positional drift. Using this modality, we have successfully found negative ions constructing distinct clusters inside nuclei. Our modality has an advantage in that chemicals contained in tiny structures that are hard to separate or purify can be directly analyzed. We believe our method will help to enrich the discussions on theoretical models of the mechanisms underlying intranuclear organelles.

## Methods

### Chemicals

4′,6-diamidino-2-phenylindole (DAPI), ultrapure water, and standard samples of nucleotides (adenine, guanine, cytosine, and thymine) were purchased from Wako Pure Chemical Industries (Tokyo, Japan).

### Preparation of animal samples

C57BL/6J mice were used in the experiments. All experiments were conducted according to protocols approved by the Animal Care and Use Committee of the Hamamatsu University School of Medicine. Mouse brains were rapidly frozen after harvest in powdered dry ice. Tissue blocks were sectioned at −16 °C using a cryostat (CM 1850; Leica Microsystems, Wetzlar, Germany) to a thickness of 10 μm. The frozen sections were thaw-mounted on indium-tin-oxide (ITO)-coated glass slides (Bruker Daltonics, Bremen, Germany). The samples were stored at −20 °C and dried at room temperature prior to the experiments. Following dehydration, the tissue sections had a thickness in the range of 2.0 ~ 2.5 μm.

### TOF-SIMS analysis

TOF-SIMS depth profiles and analyses were performed by using a PHI TRIFT V nanoTOF (ULVAC-PHI, Kanagawa, Japan) instrument. A 60 keV Au_3_^2+^ liquid metal ion gun (LMIG) was used as the primary ion source because it is suitable for detecting relatively large ions. A triple-focusing time-of-flight (TRIFT) mass analyzer provides highly sensitive measurement with a low noise level^57^. The diameter of the ion beam spot was 100 nm, and the direct current of the primary ion beam transmitted to the sample was 2 nA. For the mass calibration, CH^−^ (*m/z* 13.0), C_2_H^−^ (*m/z* 25.0), and C_4_H^−^ (*m/z* 49.0) were used. The parameters, such as the spatial resolution and direct current, were optimized to obtain good mass spectra under each experimental condition. Negative secondary ions in the *m/z* range of 0 to 1850 were obtained from 50 μm × 50 μm square regions with an image resolution of 256 × 256 pixels within 90 minutes of acquisition time. Primary ion dose density was 6.8 × 10^12^ ions/cm^2^. All spectra were acquired automatically using WinCadenceN software (ULVAC-PHI), which was used also to process TOF-SIMS data, such as the reconstruction of ion images. For further image processing, ion images were exported in a noncompressed 8-bit grayscale tiff.

### GCIB sputtering and topography analysis

Sputtering was performed by GCIB (ULVAC-PHI) after each TOF-SIMS measurement using a 10 keVAr_2500_^+^ gas cluster. The direct current delivered to the sample was 4 nA. Compared to the conventionally used C_60_ sputter beam, Ar_2500_^+^ GCIB has the advantage of avoiding contamination from the gas cluster and thus not affecting the mass spectrum^32^. Sputtering was performed on an approximately 500 μm × 500 μm square region for 3.5 minutes as one sputtering cycle. After the sample was completely sputtered out of the targeted region, we measured the precise depth profile using a DEKTAK 6M Stylus Profiler (Veeco Instruments, Plainview, NY, USA). The ion dose density of GCIB was 2.1 × 10^15^ ions/cm^2^, and 2.5 × 10^16^ ions/cm^2^ was the final dose density of the sample.

### DAPI staining and fluorescence microscopy

We performed TOF-SIMS measurement and DAPI staining on the same region of the section. Prior to the TOF-SIMS measurement and DAPI staining, enough sputtering was performed to obtain an aimed depth. First, TOF-SIMS measurements of the cell contents in a linear plane manner were performed. Second, 50 μg of DAPI powder was dissolved into 50 mL of ultrapure water, and staining was performed by incubating the sample in the solution at room temperature for 1 min. To reduce slippage and the collapse of sections, a subsequent wash was not performed, because the signal-to-noise ratio was good enough to allow us to observe the nuclei. The DAPI fluorescence was observed using an inverted microscope (TE2000, Nikon, Tokyo) equipped with a charge-coupled device camera (ORCA-ER, Hamamatsu, Shizuoka, Japan), a 40 × objective lens (Plan Fluor 40x/0.75, Nikon), and an additional intermediate 1.5 × magnification module. Fluorescence intensity over 512 × 512 pixels was acquired as 16-bit grayscale tiff images using Aqua Cosmos Software (Hamamatsu).

### Reconstruction of 3D objects and cluster size analysis

Ion images exported as 8-bit grayscale tiff images from TOF-SIMS data were processed using the ImageJ program (National Institutes of Health, Bethesda, MD, USA), which is available from the NIH website (http://rsbweb.nih.gov/ij/). The 3D objects were reconstructed using V-CAT software version 1.5 (RIKEN, Japan, http://vcad-hpsv.riken.jp/jp/release_software/V-Cat/) and 3D Viewer, a default plugin of ImageJ. The ion aggregations, which we call clusters in the present study, were analyzed using 3D Object Counter^58^, an optional plugin program for ImageJ, and areas enveloping the CN^−^-rich region were estimated using the Kalman Stack Filter plugin, which is also available from the NIH website. For these analyses, nonspecific small clusters (<5 voxels) were ignored. Colocalization of ion signals was analyzed using the Image Calculator function in ImageJ.

## Author Contributions

N.M., I.I., G.L.F., N.S., and M.S. conceived the project. I.I. and T.H. performed the experiments. N.M. and H.Y. performed the analyses. N.M., I.I., T.H. and M.S. wrote the paper.

## Additional Information

**How to cite this article**: Masaki, N. *et al*. Three-Dimensional Image of Cleavage Bodies in Nuclei Is Configured Using Gas Cluster Ion Beam with Time-of-Flight Secondary Ion Mass Spectrometry. *Sci. Rep*. **5**, 10000; doi: 10.1038/srep10000 (2015).

## Supplementary Material

Supplementary Information

Supplementary Figure S1

Supplementary Figure S2

Supplementary Figure S3

Supplementary Figure S4

## References

[b1] Dekker J., Marti-Renom M. A. & Mirny L. A. Exploring the three-dimensional organization of genomes: interpreting chromatin interaction data. Nat. Rev. Genet. 14, 390–403 (2013). 23657480 10.1038/nrg3454 PMC3874835

[b2] Levine M., Cattoglio C. & Tjian R. Looping Back to Leap Forward: Transcription Enters a New Era. Cell. 157, 13–25 (2014). 24679523 10.1016/j.cell.2014.02.009 PMC4059561

[b3] Mercer T. R. & Mattick J. S. Understanding the regulatory and transcriptional complexity of the genome through structure. Genome. Research. 23, 1081–1088 (2013). 23817049 10.1101/gr.156612.113 PMC3698501

[b4] Levesque M. J. & Raj A. Single-chromosome transcriptional profiling reveals chromosomal gene expression regulation. Nat. Meth. 10, 246–248 (2013). 10.1038/nmeth.2372 PMC4131260 23416756

[b5] Masaki N., Fujimoto K., Honda-Kitahara M., Hada E. & Sawai S. Robustness of Self-Organizing Chemoattractant Field Arising from Precise Pulse Induction of Its Breakdown Enzyme: A Single-Cell Level Analysis of PDE Expression in Dictyostelium. Biophysical journal 104, 1191–1202 (2013). 23473502 10.1016/j.bpj.2013.01.023 PMC3870794

[b6] Cedar H. & Bergman Y. Linking DNA methylation and histone modification: patterns and paradigms. Nat. Rev. Genet. 10, 295–304 (2009). 19308066 10.1038/nrg2540

[b7] Krijger P. H. L. & de Laat W. Identical cells with different 3D genomes; cause and consequences ? Current Opinion in Genetics & Development 23, 191–196 (2013). 23415810 10.1016/j.gde.2012.12.010

[b8] Jaenisch R. & Bird A. Epigenetic regulation of gene expression: how the genome integrates intrinsic and environmental signals. Nat. Genet. 33 **Suppl**, 245–254 (2003). 12610534 10.1038/ng1089

[b9] Gao F., Wei Z., Lu W. & Wang K. Comparative analysis of 4C-Seq data generated from enzyme-based and sonication-based methods. BMC. Genomics. 14, 345; 10.1186/1471-2164-14-345 (2013). 23705979 PMC3679908

[b10] de Wit E. . The pluripotent genome in three dimensions is shaped around pluripotency factors. Nature 501, 227–231 (2013). 23883933 10.1038/nature12420

[b11] Mao Y. S., Zhang B. & Spector D. L. Biogenesis and function of nuclear bodies. Trends in Genetics 27, 295–306 (2011). 21680045 10.1016/j.tig.2011.05.006 PMC3144265

[b12] Gao L. . Noninvasive Imaging beyond the Diffraction Limit of 3D Dynamics in Thickly Fluorescent Specimens. Cell. 151, 1370–1385 (2012). 23217717 10.1016/j.cell.2012.10.008 PMC3615549

[b13] Wu Y. . Spatially isotropic four-dimensional imaging with dual-view plane illumination microscopy. Nat. Biotech. 31, 1032–1038 (2013). 10.1038/nbt.2713 PMC4105320 24108093

[b14] Gebhardt J. C. M. . Single-molecule imaging of transcription factor binding to DNA in live mammalian cells. Nat. Meth. 10, 421–426 (2013). 10.1038/nmeth.2411 PMC3664538 23524394

[b15] Susaki Etsuo A. . Whole-Brain Imaging with Single-Cell Resolution Using Chemical Cocktails and Computational Analysis. Cell. 157, 726–739 (2014). 24746791 10.1016/j.cell.2014.03.042

[b16] Schermelleh L., Heintzmann R. & Leonhardt H. A guide to super-resolution fluorescence microscopy. The Journal of Cell. Biology 190, 165–175 (2010). 20643879 10.1083/jcb.201002018 PMC2918923

[b17] Heuser J. Whatever happened to the ‘microtrabecular concept’? Biology of the cell/under the auspices of the European Cell Biology Organization 94, 561–596 (2002). 12732437 10.1016/s0248-4900(02)00013-8

[b18] Hermann R., Walther P. & Müller M. Immunogold labeling in scanning electron microscopy. Histochem. Cell. Biol. 106, 31–39 (1996). 8858365 10.1007/BF02473200

[b19] Hanrieder J., Malmberg P., Lindberg O. R., Fletcher J. S. & Ewing A. G. Time-of-Flight Secondary Ion Mass Spectrometry Based Molecular Histology of Human Spinal Cord Tissue and Motor Neurons. Analytical Chemistry 85, 8741–8748 (2013). 23947367 10.1021/ac401830m PMC3871880

[b20] Zinner E. Laboratory Analysis of Stardust. Analytical Chemistry 85, 1264–1270 (2012). 23231704 10.1021/ac303052p

[b21] Cabral R. A. . Anomalous sulphur isotopes in plume lavas reveal deep mantle storage of Archaean crust. Nature 496, 490–493 (2013). 23619695 10.1038/nature12020

[b22] Jung K.-W. . Quantitative Compositional Profiling of Conjugated Quantum Dots with Single Atomic Layer Depth Resolution via Time-of-Flight Medium-Energy Ion Scattering Spectroscopy. Analytical Chemistry 86, 1091–1097 (2013). 24350771 10.1021/ac402753j

[b23] Passarelli M. K., Ewing A. G. & Winograd N. Single-Cell Lipidomics: Characterizing and Imaging Lipids on the Surface of Individual Aplysia californica Neurons with Cluster Secondary Ion Mass Spectrometry. Analytical Chemistry 85, 2231–2238 (2013). 23323749 10.1021/ac303038j PMC3867296

[b24] Senyo S. E. . Mammalian heart renewal by pre-existing cardiomyocytes. Nature 493, 433–436 (2013). 23222518 10.1038/nature11682 PMC3548046

[b25] Fisher G. L., Belu A. M., Mahoney C. M., Wormuth K. & Sanada N. Three-Dimensional Time-of-Flight Secondary Ion Mass Spectrometry Imaging of a Pharmaceutical in a Coronary Stent Coating as a Function of Elution Time. Analytical Chemistry 81, 9930–9940 (2009). 19919043 10.1021/ac901587k

[b26] Winograd N. . Improvements in SIMS continue: Is the end in sight ? Applied Surface Science 252, 6836–6843 (2006).

[b27] Russo M. F. & Garrison B. J. Mesoscale Energy Deposition Footprint Model for Kiloelectronvolt Cluster Bombardment of Solids. Analytical Chemistry 78, 7206–7210 (2006). 17037922 10.1021/ac061180j

[b28] Fletcher J. S., Rabbani S., Henderson A., Lockyer N. P. & Vickerman J. C. Three-dimensional mass spectral imaging of HeLa-M cells – sample preparation, data interpretation and visualisation. Rapid Communications in Mass Spectrometry 25, 925–932 (2011). 21416529 10.1002/rcm.4944

[b29] Brison J. . TOF-SIMS 3D Imaging of Native and Non-Native Species within HeLa Cells. Analytical Chemistry 85, 10869–10877 (2013). 24131300 10.1021/ac402288d PMC3889863

[b30] Bich C. . Argon Cluster Ion Source Evaluation on Lipid Standards and Rat Brain Tissue Samples. Analytical Chemistry 85, 7745–7752 (2013). 23875833 10.1021/ac4009513

[b31] Matsuo J. . SIMS with highly excited primary beams for molecular depth profiling and imaging of organic and biological materials. Surface and Interface Analysis 42, 1612–1615 (2010).

[b32] Rabbani S., Barber A. M., Fletcher J. S., Lockyer N. P. & Vickerman J. C. TOF-SIMS with Argon Gas Cluster Ion Beams: A Comparison with C60+. Analytical Chemistry 83, 3793–3800 (2011). 21462969 10.1021/ac200288v

[b33] Miyayama T., Sanada N., Bryan S. R., Hammond J. S. & Suzuki M. Removal of Ar+ beam-induced damaged layers from polyimide surfaces with argon gas cluster ion beams. Surface and Interface Analysis 42, 1453–1457 (2010).

[b34] Lee S. . Ultra-thin resistive switching oxide layers self-assembled by field-induced oxygen migration (FIOM) technique. Sci. Rep. 4, 6871; 10.1038/srep06871 (2014). 25362933 PMC4217097

[b35] Kang D.-Y. & Moon J. H. Lithographically Defined Three-dimensional Pore-patterned Carbon with Nitrogen Doping for High-Performance Ultrathin Supercapacitor Applications. Sci. Rep. 4, 5392; 10.1038/srep05392 (2014). 24953307 PMC4066249

[b36] Sjövall P., Johansson B. & Lausmaa J. Localization of lipids in freeze-dried mouse brain sections by imaging TOF-SIMS. Applied Surface Science 252, 6966–6974 (2006).

[b37] Petit V. W. . Multimodal Spectroscopy Combining Time-of-Flight-Secondary Ion Mass Spectrometry, Synchrotron-FT-IR, and Synchrotron-UV Microspectroscopies on the Same Tissue Section. Analytical Chemistry 82, 3963–3968 (2010). 20387890 10.1021/ac100581y

[b38] Touboul D., Brunelle A., Halgand F., De La Porte S. & Laprévote O. Lipid imaging by gold cluster time-of-flight secondary ion mass spectrometry: application to Duchenne muscular dystrophy. Journal of Lipid Research 46, 1388–1395 (2005). 15834124 10.1194/jlr.M500058-JLR200

[b39] Cillero-Pastor B., Eijkel G., Kiss A., Blanco F. J. & Heeren R. M. A. Time-of-Flight Secondary Ion Mass Spectrometry-Based Molecular Distribution Distinguishing Healthy and Osteoarthritic Human Cartilage. Analytical Chemistry 84, 8909–8916 (2012). 22950553 10.1021/ac301853q

[b40] Sugiura Y. . Visualization of the cell-selective distribution of PUFA-containing phosphatidylcholines in mouse brain by imaging mass spectrometry. Journal of Lipid Research 50, 1776–1788 (2009). 19417221 10.1194/jlr.M900047-JLR200 PMC2724791

[b41] Sjövall P. . Imaging of Distribution of Topically Applied Drug Molecules in Mouse Skin by Combination of Time-of-Flight Secondary Ion Mass Spectrometry and Scanning Electron Microscopy. Analytical Chemistry 86, 3443–3452 (2014). 24568123 10.1021/ac403924w

[b42] Benabdellah F. . Mass spectrometry imaging of rat brain sections: nanomolar sensitivity with MALDI versus nanometer resolution by TOF–SIMS. Analytical and Bioanalytical Chemistry 396, 151–162 (2010). 19711060 10.1007/s00216-009-3031-2

[b43] Yang K., Zhao Z., Gross R. W. & Han X. Identification and Quantitation of Unsaturated Fatty Acid Isomers by Electrospray Ionization Tandem Mass Spectrometry: A Shotgun Lipidomics Approach. Analytical Chemistry 83, 4243–4250 (2011). 21500847 10.1021/ac2006119 PMC3105155

[b44] Ulmann L., Mimouni V., Roux S., Porsolt R. & Poisson J. P. Brain and hippocampus fatty acid composition in phospholipid classes of aged-relative cognitive deficit rats. Prostaglandins, Leukotrienes and Essential Fatty Acids. 64, 189–195 (2001). 10.1054/plef.2001.0260 11334555

[b45] Carrié I., Clément M., de Javel D., Francès H. & Bourre J.-M. Specific phospholipid fatty acid composition of brain regions in mice: effects of n–3 polyunsaturated fatty acid deficiency and phospholipid supplementation. Journal of Lipid Research 41, 465–472 (2000). 10706594

[b46] Konishi Y. & Setou M. Tubulin tyrosination navigates the kinesin-1 motor domain to axons. Nat. Neurosci. 12, 559–567 (2009). 19377471 10.1038/nn.2314

[b47] Kawakami R. . Visualizing hippocampal neurons with in vivo two-photon microscopy using a 1030 nm picosecond pulse laser. *Sci*. Rep. 3, 1014; 10.1038/srep01014 (2013). PMC3553458 23350026

[b48] Schul W., van der Kraan I., Matera A. G., van Driel R. & de Jong L. Nuclear Domains Enriched in RNA 3′-processing Factors Associate with Coiled Bodies and Histone Genes in a Cell Cycle–dependent Manner. Molecular Biology of the Cell. 10, 3815–3824 (1999). 10564273 10.1091/mbc.10.11.3815 PMC25681

[b49] Martone M. E., Pollock J. A., Jones Y. Z. & Ellisman M. H. Ultrastructural localization of dendritic messenger RNA in adult rat hippocampus. The Journal of neuroscience : the official journal of the Society for Neuroscience 16, 7437–7446 (1996). 8922399 10.1523/JNEUROSCI.16-23-07437.1996 PMC6579092

[b50] Chen H.-C., Lin W.-C., Tsay Y.-G., Lee S.-C. & Chang C.-J. An RNA Helicase, DDX1, Interacting with Poly(A) RNA and Heterogeneous Nuclear Ribonucleoprotein K. Journal of Biological Chemistry 277, 40403–40409 (2002). 12183465 10.1074/jbc.M206981200

[b51] Norland S., Fagerbakke K. M. & Heldal, M. Light element analysis of individual bacteria by x-ray microanalysis. Applied and Environmental Microbiology 61, 1357–1362 (1995). 16534992 10.1128/aem.61.4.1357-1362.1995 PMC1388410

[b52] Krafft C., Knetschke T., Siegner A., Funk R. H. W. & Salzer R. Mapping of single cells by near infrared Raman microspectroscopy. Vibrational Spectroscopy 32, 75–83 (2003).

[b53] Zimber A., Nguyen Q.-D. & Gespach C. Nuclear bodies and compartments: functional roles and cellular signalling in health and disease. Cellular Signalling 16, 1085–1104 (2004). 15240004 10.1016/j.cellsig.2004.03.020

[b54] Koberna K. . Ribosomal genes in focus: new transcripts label the dense fibrillar components and form clusters indicative of “Christmas trees” in situ. The Journal of Cell Biology 157, 743–748 (2002). 12034768 10.1083/jcb.200202007 PMC2173423

[b55] Schul W. . The RNA 3’ cleavage factors CstF 64 kDa and CPSF 100 kDa are concentrated in nuclear domains closely associated with coiled bodies and newly synthesized RNA. The EMBO journal 15, 2883–2892 (1996). 8654386 PMC450226

[b56] Li L. . Dynamic Nature of Cleavage Bodies and Their Spatial Relationship to DDX1 Bodies, Cajal Bodies, and Gems. Molecular Biology of the Cell. 17, 1126–1140 (2006). 16371507 10.1091/mbc.E05-08-0768 PMC1382303

[b57] Schueler B. W. Microscope imaging by time-of-flight secondary ion mass spectrometry. Microsc. Microanal. Microstruct. 3, 119–139 (1992).

[b58] Bolte S. & CordeliÈRes F. P. A guided tour into subcellular colocalization analysis in light microscopy. Journal of Microscopy 224, 213–232 (2006). 17210054 10.1111/j.1365-2818.2006.01706.x

