# Supplementary material for: Three-Dimensional Image of Cleavage Bodies in Nuclei Is Configured Using Gas Cluster Ion Beam with Time-of-Flight Secondary Ion Mass Spectrometry
Source: Sci Rep. 2015 May 11;5:10000. doi: 10.1038/srep10000 (PMC4426704; doi:10.1038/srep10000)
Supplement: Supplementary Information [file srep10000-s1.pdf]

Supplementary Information

Three-Dimensional Image of Cleavage Bodies in Nuclei Is Configured Using Gas Cluster Ion Beam with Time-of-Flight Secondary Ion Mass Spectrometry

Noritaka Masaki, Itsuko Ishizaki, Takahiro Hayasaka, Gregory L. Fisher, Noriaki Sanada, Hideo Yokota, and Mitsutoshi Setou

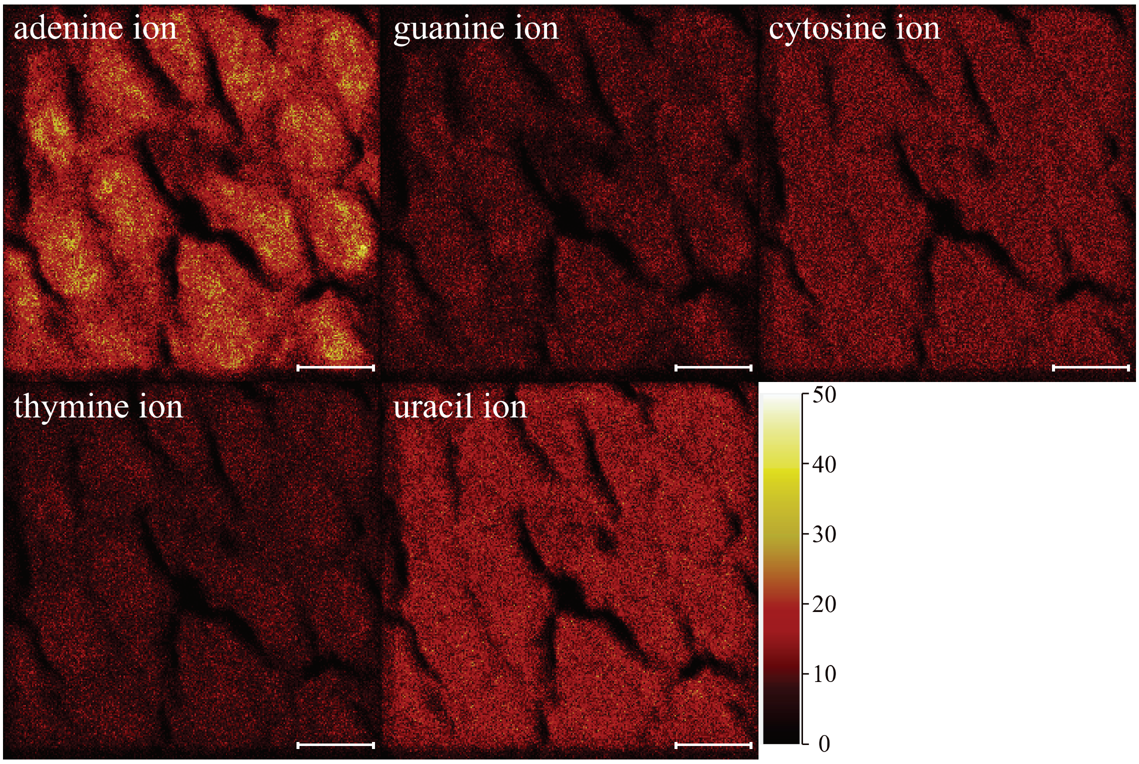

Figure S1 | Ion images of nucleic acids. Signals from adenine, guanine, cytosine, thymine, and uracil were integrated from all the depth planes and shown in pseudo-color scale. Scale bar represents 10 μm.

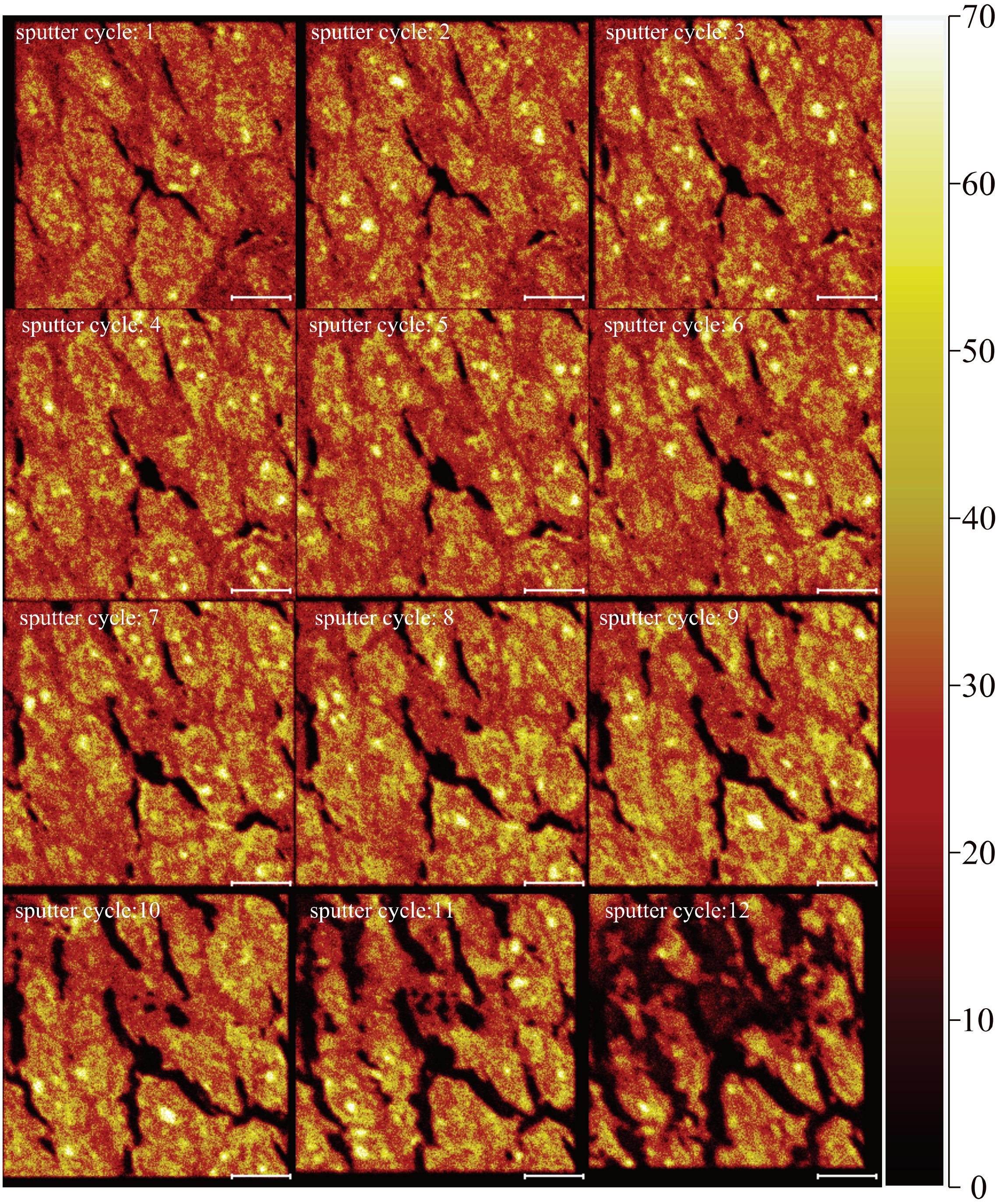

Figure S2 | Ion images of PO_3^- reconstructed for each depth plane. Ion images were reconstructed for sputter cycle from 1 to 12. PO_3^- also shows distribution at CN^--rich region. To improve distribution other than clusters, signal intensity higher than 70 is saturated. Signal intensity is represented in a pseudo color scale and scale bar represents 10 μm.

Supplementary Movies

Four movies exhibiting rotational view of 3 dimensional (3D) objects are available via the website, http://www.nature.com/scientificreports. These movies are provided as H.264 format (*.mp4) files.

Movie S1 | Rotational view of whole 3D objects with cell bodies shown in Fig. 4a. Loci of CN^-, PO_3^-, and adenine ions are shown as binarized images of blue, green, and red, respectively. Cell bodies are also shown as total ion signals with a relative intensity in 8-bit yellow scale.

Movie S2 | Rotational view of whole 3D objects without cell bodies shown in Fig. 4b. Loci of CN^-, PO_3^-, and adenine ions are shown as binarized images of blue, green, and red, respectively.

Movie S3 | Rotational view corresponding to the single CN^--rich region shown in Fig. 4c. Loci of CN^-, PO_3^-, and adenine ions are shown as binarized images of blue, green, and red, respectively. The cell body is included and shown as total ion signals with a relative intensity in 8-bit yellow scale.

Movie S4 | Rotational view corresponding to the single CN^--rich region shown in Fig. 4d. Loci of CN^-, PO_3^-, and adenine ions are shown as binarized images of blue, green, and red, respectively. To improve visualization, transparency of CN^- rich region was increased.
